# High Prevalence of Co-Infection with Multiple Torque Teno Sus Virus Species in Italian Pig Herds

**DOI:** 10.1371/journal.pone.0113720

**Published:** 2014-11-20

**Authors:** Sylvain Blois, Francesca Mallus, Manuele Liciardi, Cristian Pilo, Tania Camboni, Lisa Macera, Fabrizio Maggi, Aldo Manzin

**Affiliations:** 1 Department of Biomedical Sciences, Clinical Microbiology and Virology Unit, University of Cagliari Medical School, Cagliari, Italy; 2 Istituto Zooprofilattico Sperimentale Sardegna, Department of Cagliari, Cagliari, Italy; 3 Virology Unit, Pisa University Hospital, Pisa, Italy; Virginia Polytechnic Institute and State University, United States of America

## Abstract

Torque teno viruses (TTVs) are a large group of vertebrate-infecting small viruses with circular single-stranded DNA, classified in the *Anelloviridae* family. In swine, two genetically distinct species, *Torque teno sus virus 1a* (TTSuV1a) and *1b* (TTSuV1b) are currently grouped into the genus *Iotatorquevirus*. More recently, a novel Torque teno sus virus species, named *Torque teno sus virus k2b* (TTSuVk2b), has been included with *Torque teno sus virus k2a* (TTSuVk2a) into the genus *Kappatorquevirus*. In the present study, TTSuV1 (TTSuV1a and TTSuV1b), TTSuVk2a and TTSuVk2b prevalence was evaluated in 721 serum samples of healthy pigs from Sardinian farms, insular Italy. This is the largest study to date on the presence of TTSuV in healthy pigs in Italy. The global prevalence of infection was 83.2% (600/721), being 62.3% (449/721), 60.6% (437/721), and 11.5% (83/721) the prevalence of TTSuV1, TTSuVk2a and TTSuVk2b, respectively. The rate of co-infection with two and/or three species was also calculated, and data show that co-infections were significantly more frequent than infections with single species, and that TTSuV1+TTSuVk2a double infection was the prevalent combination (35.4%). Quantitative results obtained using species-specific real time-qPCR evidenced the highest mean levels of viremia in the TTSuV1 subgroup, and the lowest in the TTSuVk2b subgroup. Interestingly, multiple infections with distinct TTSuV species seemed to significantly affect the DNA load and specifically, data highlighted that double infection with TTSuVk2a increased the viral titers of TTSuV1, likewise the co-infection with TTSuVk2b increased the titers of TTSuVk2a.

## Introduction

Anelloviruses (AVs), recently classified in the new family *Anelloviridae*, are a large group of non-enveloped viruses which contain a single-stranded, negative sense, circular DNA genome; because of their heterogeneity, AVs are divided into 11 genera, each one with various numbers of species (ICTV, International Committee on Taxonomy of Viruses). AVs are similar to circoviruses [1], in particular the porcine circovirus type 2 (PCV2) [2], which causes a severe multisystemic wasting syndrome post-weaning (PMWS, porcine multisystemic wasting syndrome) and immunosuppression, and the avian anemia virus (CAV, chicken anemia virus), responsible of a severe hematological diseases in poultry. Following the discovery of Torque teno virus (TTV) in humans [3], AVs were identified also in pigs, cattle, sheep, dogs, cats, chimpanzees and other animals [4], [5], [6]. Anelloviruses from different species share a similar genomic organization but a low sequence homology. Except for a short conserved nucleotide sequence located in the 5′-untranslated region near the Tata box, AVs from different species show great genetic diversity among genera (over 56% in ORF 1) and within genus (35 to 55%) [7], [8]. Further, although the genomes of some animal and human AVs cluster together, they are quite divergent in sequence composition and genome length [9]. Interactions between AVs and their host are mostly unknown, and their high prevalence worldwide raised doubts on their actual pathogenic potential up to the point that some researchers have considered TTV a component of the human microbiota [10]. Further studies are needed to elucidate this issue thoroughly.

The observation that some pet animals (cats and dogs) and livestock (cattle, sheep, pigs, and poultry) are infected with AVs identical or very similar to human AVs (human and sus TTV [TTSuV] exhibit nearly 50% nucleotide identity) has led to the hypothesis that interspecies transmission is a fairly common event. Recent molecular surveys carried out in different geographic areas, found 24 to 100% pigs positive for TTSuV [11], [12], [13], [14] and, like in human TTVs, genetic variability was high and co-infection with multiple viral subtypes frequent [5], [15]. Two swine-infecting species, *Torque teno sus virus 1a* (TTSuV1a) and *Torque teno sus virus 1b* (TTSuV1b) are now classified in the genus *Iotatorquevirus* [16]. More recently, a novel Torque teno sus virus species named *Torque teno sus virus k2b* (TTSuVk2b), classified with *Torque teno sus virus k2a* (TTSuVk2a) in the *Kappatorquevirus* genus of the family *Anelloviridae*, has been described in pig sera [14]. TTSuVs are ubiquitous and distributed worldwide [17], both in domestic pigs and wild boars [18], [19]. TTSuVs are likely transmitted horizontally, as viral DNA has been detected in sera, feces, semen and nasal secretions [6], [20], [21]. TTSuV DNA has also been found in colostrum and stillborn, suggesting that the vertical and transplacental/intra-uterine transmissions are important routes of dissemination [22], [23]. Infection with TTSuV occurs early in life and leads to a progressive persistent infection, and viral load appears to increase with the age of the animals [24], [25]. Nevertheless, the role of TTV infection in host animals is still poorly understood [26], [27]: although most infected animals, if not all, are apparently healthy, it is possible that TTSuVs contribute directly and/or through interaction with other pathogens to cause some important ailments [12], [28]. As reported for TTV infection in humans, TTSuVs could act as superimposed agents or trigger diseases that progressively impair the immune system [29]. Finally, the contribution of animal AVs to the epidemiology of TTVs in humans is still unknown. In particular, TTSuV has been proposed as a model to evaluate the dynamics and the effects of global trade on viral heterogeneity and to understand how live pigs movement affect virus population and evolution [30].

So far, the presence of TTSuVs in Italian pig herds has been poorly investigated [13] as well as the molecular epidemiology of the infection within swine farms. This is the first extensive study on presence, quantitation and genotypic characterization of TTSuVs in Sardinia, Italy; here we analyzed more than 700 sera from commercial pigs and found a high prevalence of infection, in particular often with multiple species simultaneously, and variable levels of circulating viruses.

## Materials and Methods

### Samples

Seven hundred and twenty one domestic swine sera (males *n* = 133, females *n* = 588; animals under twelve months of age *n* = 113, animals over twelve months of age *n* = 608) were aliquots of a larger stored collection of sera previously analyzed within the regional monitoring plan for African swine fever (ASF) and Classical swine fever (CSF) enforced in Sardinia, in compliance with the European Community requirements. Sera had been collected between 2012 and 2013 from clinically healthy pigs by staff veterinarians of the Italian Public Health System, according to Decrees No. 9, 16.05.2007, No. 1567/decA/23, and No. 20/09.07.2013 of the “Assessorato dell’Igiene e Sanità e dell’Assistenza Sociale, Regione Autonoma della Sardegna” at farms located in central and southern Sardinia. No animal was sacrificed for the purpose of this study, and swine sera were collected according to the “Guida al prelievo e recapito dei campioni”, Istituto Zooprofilattico Sperimentale della Sardegna “G. Pegreffi”, Rev. 25.05.2012. Sera were stored at −80°C until use.

### DNA extraction

DNA extraction was performed from 100 µl of each serum using the QIAamp DNA Mini kit (Qiagen) according to the manufacturer’s instructions. DNA was eluted in 100 µl of elution buffer and stored at −20°C until use. All DNA extraction procedures included a negative control, containing only PBS as extraction substrate.

### Species-specific PCR and real time-qPCR assays for TTSuV DNA detection and quantification

PCR assays specific for TTSuV species 1 (both genotypes TTSuV1a and TTSuV1b), k2a and k2b were used to detect the presence of viral DNA. Briefly, for TTSuV1 detection, 25 µl of the PCR reaction contained 3 µl of serum DNA, 10 pmol of each primer TTSuV1_F (5′-CGGGTTCAGGAGGCTCAAT-3′) and TTSuV1_R (5′-GCCATTCGGAACTGCACTTACT-3′) [18] were used in a buffer containing 400 µM dNTPs, 2 mM of MgCl_2_ and 0.75 U of PolyTaq DNA polymerase (Polymed). After an initial denaturation step for 5 min at 94°C, the amplification was performed by 50 cycles of 15 s at 94°C, 20 s at 54°C, 30 s at 72°C followed by a final extension step for 5 min at 72°C. For TTSuVk2a detection, PCR reactions were carried out as described above with an annealing step of 20 s at 56°C using the primer pair TTSuV2_F (5′-TCATGACAGGGTTCACCGGA-3′) and TTSuV2_R (5′-CGTCTGCGCACTTACTTATATACTCTA-3′) [18]. For TTSuVk2b detection, the annealing step was performed for 20 s at 62°C using primers TTSuV-all-F1 (5′-CGAATGGCTGAGTTTATGCCGC-3′) and TTV3-361R (5′-TTCGCTGTGACTGGCGTCTC-3′) [14]. The presence of TTSuV1, TTSuVk2a and TTSuVk2b DNA was determined respectively by the detection of a 305 bp, 250 bp and 90 bp fragment, on a 2% SYBR safe stained TAE-agarose gel (Life Technologies).

Specific real time quantitative PCR (real time-qPCR) assays were performed on positive samples to determine the viral DNA load. Briefly, 20 µl PCR mixture contained 10 µl of SsoAdvanced Universal SYBR Green Supermix (Bio-Rad), 8 pmol of each primer (PTTV1F and PTTV1R for TTSuV1; TTV2-215F and TTV2-360R for TTSuVk2a; TTSuV-all-F1 and TTV3-361R for TTSuVk2b) [14], [31] and 5 µl of DNA elution were used for each reaction. qPCR reactions consisted of an initial denaturation step of 30 s at 95°C, followed by 50 cycles of 5 s at 95°C, 20 s at 59.5°C for TTSuV1, 20 s at 56°C for TTSuVk2a or 10 s at 62°C for TTSuVk2b. The specificity of each PCR was determined by the melting curve analysis increasing the temperature from 65°C to 95°C with 0.5°C increments. Fluorescence was measured during each extension step and melting curve analysis. Real time-qPCR was performed on a CFX96 Real-Time System instrument (Bio-Rad) using the Bio-Rad CFX manager software, version 3.1.

The copy number of TTSuV1, TTSuVk2a and TTSuVk2b DNA per ml of serum was determined in reference to a standard curve ranging from 10^6^ to 10 copies prepared in duplicate by ten-fold serial dilution of plasmids TTV1_G26 (TTSuV1 isolate TTV1_G26), TTV2_GE9 2 (TTSuVk2a isolate TTV2_GE9), and TTSuVk2b (TTSuVk2b isolate 38E05), respectively [14], [32]. Finally, the mean log_10_ DNA copy numbers per ml of serum was used to compare data. Limit of detection was 10 copies per reaction for each qPCR assay.

### Sequencing

The specificity of reactions was confirmed by the sequence analysis of randomly selected amplicons (5 to 10 for each assay). PCR products were purified using QIAquick PCR purification kit (Qiagen). Sequencing reactions were bidirectionally carried out using BigDye Terminator v3.1 cycle sequencing kit (Applied Biosystems) and run using ABI Prism 3100 Genetic analyzer (Applied Biosystems). The nucleotide sequences were compared with reference sequences for each species available in GenBank using the online program NCBI BLAST.

### Statistical analysis

TTSuV prevalence-related data were analyzed using the Chi-square test. TTSuV viral load expressed as log_10_ DNA copies per ml was normally distributed. Differences in mean viral titers among species were analyzed using the one-way analysis of variance (ANOVA) or unpaired Student’s *t* test when appropriate. Values of p<0.05 were accepted as significant. The statistical analyses were performed using Prism software (GraphPad).

## Results

### TTSuV DNA prevalence in Sardinian domestic pig sera

TTSuV prevalence in Sardinian pig herds was evaluated by detection of TTSuV1, k2a and k2b DNA in 721 sera using species-specific PCR assays. Results are summarized in Table 1. Regardless the species, TTSuV DNA was detected in the majority of tested sera, as the global prevalence was 83.2% (600/721). No significant difference was found between the overall prevalence (single and/or co-infections) of TTSuV1 and TTSuVk2a (p = 0.5517), being 62.3% (449/721) and 60.6% (437/721), respectively, whereas the prevalence of TTSuVk2b, being 11.5% (83/721), was significantly lower (p<0.0001). Similar differences were observed within gender and age groups. Notable, when comparing overall prevalence between genders and between age groups, no significant difference was found between males and females, while the TTSuVk2b infection rate was significantly higher in younger animals (18.6%, 21/113) than in older pigs (10.2%, 62/608) (p = 0.0162) (data not shown).

**Table 1:** Prevalence of TTSuV DNA in 721 sera of Italian domestic pigs.

	Overall infections (%)	Single infections (%)	Co-infections (%)
**TTSuV1**	449 (62.3)∧	147 (20.4)	-
**TTSuVk2a**	437 (60.6)∧	115 (16.0)	-
**TTSuVk2b**	83 (11.5)	11 (1.5)	-
**TTSuV1+k2a**	-	-	255 (35.4)
**TTSuV1+k2b**	-	-	5 (0.7)
**TTSuVk2a+k2b**	-	-	25 (3.5)
**TTSuV1+k2a+k2b**	-	-	42 (5.8)
**Total**	600 (83.2)	273 (37.9)	327 (45.4)

∧Refers only to non-significant differences (p>0.05).

Considering the single infections, 37.9% (273/721) of the sera was found positive. Interestingly, the most prevalent species was TTSuV1 (20.4%), followed by TTSuVk2a (16%) and TTSuVk2b (1.5%). The prevalence was significantly different from each other (p<0.05). Instead, within gender and age groups, no significant difference was found between TTSuV1 and TTSuVk2a. Moreover, when comparing the prevalence of single infections between genders and between age groups, no differences were observed (p>0.05) (data not shown).

Furthermore, our data indicate that the rate of co-infections (45.4%, 327/721) was significantly higher than that of single infections (p<0.005). In particular, the TTSuV1+TTSuVk2a co-infection was the prevalent combination (35.4%) followed by the TTSuV1+TTSuVk2a+TTSuVk2b (5.8%), TTSuVk2a+TTSuVk2b (3.5%), and TTSuV1+TTSuVk2b (0.7%) co-infections. The frequencies were significantly different from each other (p<0.05) (Table 1).

Thus, TTSuV1 and TTSuVk2a were mainly found in combination rather than in single infections or in association with TTSuVk2b. Likewise, TTSuVk2b was mostly detected in the TTSuV1+TTSuVk2a+TTSuVk2b triple infection rather than in single infections or with one of the two other species.

The same observation was made within age groups and within genders, except that the TTSuV1 prevalence in single and co-infection with TTSuVk2a was not significantly different in younger animals and the TTSuVk2b prevalence in single and co-infections was not significantly different in males. Moreover, the triple infection frequency was significantly higher in younger (12.4%) than in older animals (4.6%) (p<0.005) (data not shown).

### Viral DNA load determination

**Figure 1:**
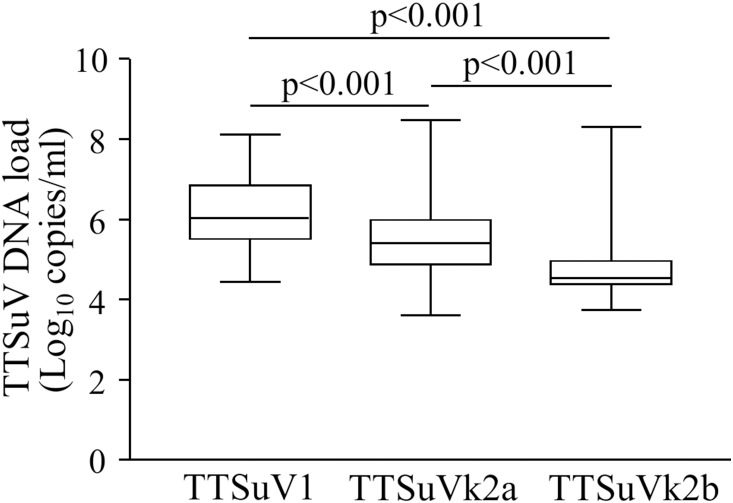
Box plots for TTSuV DNA load in the overall infections. Significant differences in mean viral load are indicated by p values.

**Figure 2:**
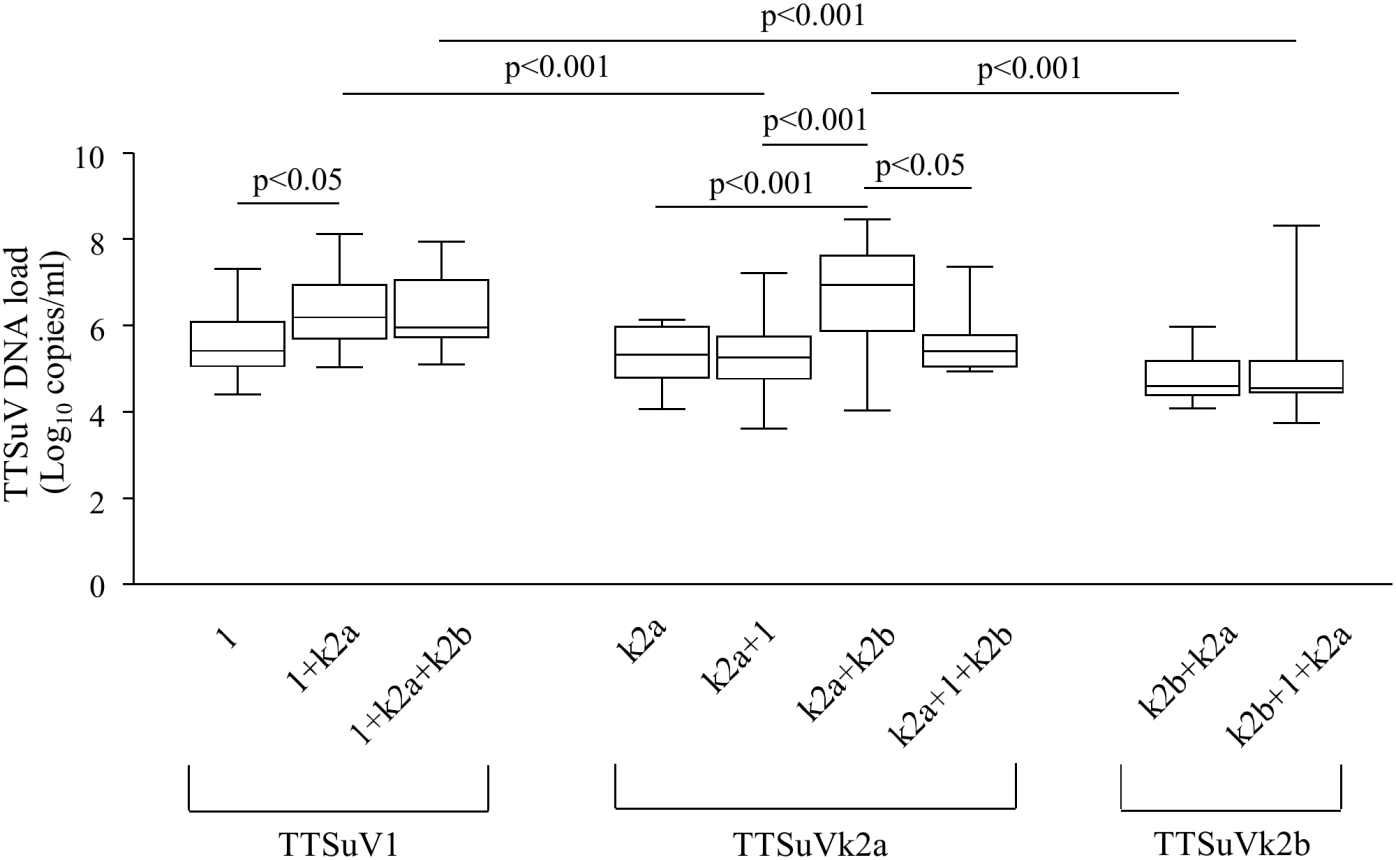
Box plots for TTSuV DNA load in the single and co-infections. 1: TTSuV1; k2a: TTSuVk2a; k2b: TTSuVkb2. Significant differences in mean viral load are indicated by p values.

The copy numbers per ml of serum of TTSuV1, TTSuVk2a and TTSuVk2b DNA were quantified by species-specific real time-qPCR. In the overall infections (single and/or co-infections), viral DNA load was in the range of 4.44–8.12 (mean 6.18±SD 0.83) log_10_ copies per ml of serum for TTSuV1, 3.62–8.47 (mean 5.50±SD 0.94) log_10_ copies per ml of serum for TTSuVk2a, and 3.75–8.31 (mean 4.73±SD 0.83) log_10_ per ml of serum for TTSuVk2b, respectively. 7.6% of the TTSuVk2a-positive samples and 3.3% of the TTSuVk2b-positive samples were under the limit of quantification (<3.3 log_10_ per ml of serum). Differences in viral DNA load among the three species were statistically significant (p<0.001) (Fig. 1). On the contrary, in single infections the difference between TTSuV1 (mean 5.65±SD 0.78 log_10_ per ml of serum) and TTSuVk2a (mean 5.31±SD 0.66 log_10_ per ml of serum) viral load was not statistically significant (Fig. 2).

Interestingly, when comparing the viral load between single and co-infections, the TTSuV1 titers were significantly higher in the double infection with TTSuVk2a (mean 6.34±SD 0.79 log_10_ per ml of serum) (p<0.05), whereas the TTSuVk2a titers did not differ (mean 5.31±SD 0.83 log_10_ per ml of serum). Noteworthy, the TTSuVk2a DNA load significantly increased in the TTSuVk2a+TTSuVk2b co-infection (mean 6.59±SD 1.23 log_10_ per ml of serum) (p<0.001), but not in the triple infection (mean 5.62±SD 0.76 log_10_ per ml of serum) (Fig. 2). With respect to the TTSuV1 DNA load in the TTSuV1+TTSuVk2b co-infection the statistical analysis was not carried out due to the limited number of sera, however, in the triple infection (mean 6.25±SD 0.80 log_10_ per ml of serum) TTSuV1 titers did not vary significantly.

Within the TTSuV1+TTSuVk2a co-infection, the TTSuV1 titers were significantly higher than that of TTSuVk2a (p<0.001). Otherwise, within the triple infection the viral load of TTSuV1 and TTSuVk2a did not significantly differ (p>0.05). Similarly, within the TTSuVk2a+TTSuVk2b co-infection, the TTSuVk2a titers (mean 6.59±SD 1.23 log_10_ per ml of serum) were significantly higher than that of TTSuVk2b (mean 4.75±SD 0.51 log_10_ per ml of serum) (p<0.001), but no significant difference was observed within the triple infection (p>0.05). TTSuVk2b DNA load did not differ significantly between TTSuVk2a+TTSuVk2b co-infection and triple infection (mean 4.90±SD 1.19 log_10_ per ml of serum) (Fig. 2).

**Figure 3:**
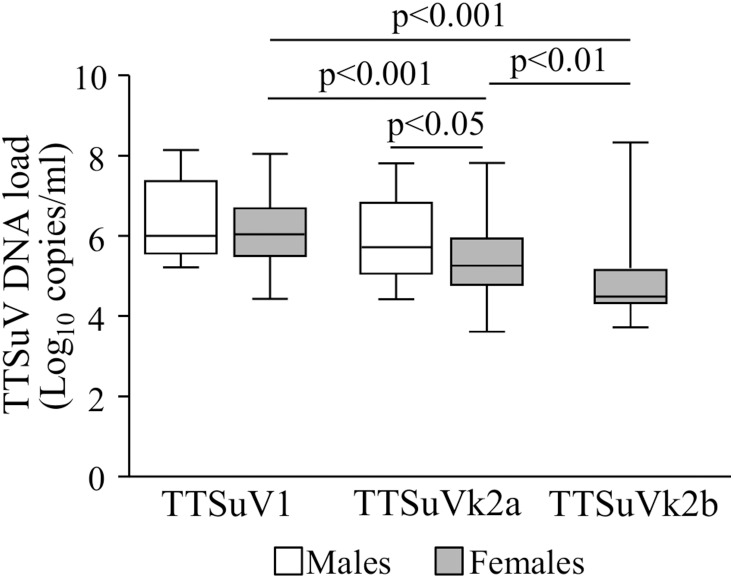
Box plots TTSuV DNA load in the overall infections of male and female pigs. Significant differences in mean viral load are indicated by p values.

**Figure 4:**
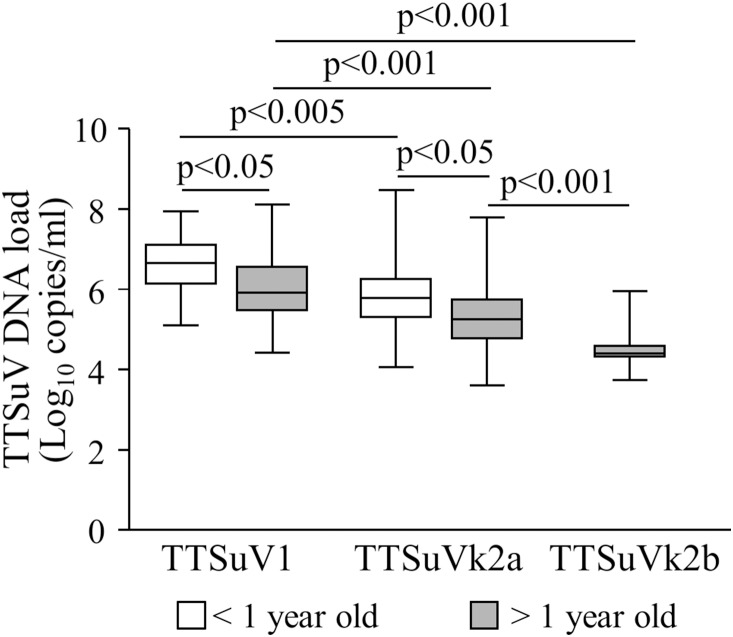
Box plots TTSuV DNA load in the overall infections of <1 year and >1 year old animals. Significant differences in mean viral load are indicated by p values.

TTSuV DNA load in the overall infections was also compared within and between genders (Fig. 3), as well as within and between age groups (Fig. 4). The statistical analysis was possible only between the TTSuV1 and TTSuVk2a infections due to the limited number of TTSuVk2b-positive samples in males and in young animals. In contrast to what observed in females, the titers of TTSuV1 (mean 6.45±SD 0.98 log_10_ per ml of serum) and TTSuVk2a (mean 5.91±SD 0.97 log_10_ per ml of serum) were not different among males (p>0.05) (Fig. 3). No statistical difference was evidenced for TTSuV1 between genders, whereas TTSuVk2a load was higher in males than in females (mean 5.38±SD 0.89 log_10_ per ml of serum) (p<0.05) (Fig. 3).

When analyzing the age groups, TTSuVs DNA titers were statistically different from each other (Fig. 4). Interestingly, the TTSuV1 and TTSuVk2a titers in the overall infections were significantly higher in younger animals (TTSuV1 mean 6.60±SD 0.71 log_10_ per ml of serum, and TTSuVk2a mean 5.82±SD 0.97 log_10_ per ml of serum) than in older swine (TTSuV1 mean 6.07±SD 0.82 log_10_ per ml of serum, and TTSuVk2a mean 5.38±SD 0.91 log_10_ per ml of serum) (p<0.05) (Fig. 4), while in the co-infections no significant difference was evidenced between younger (TTSuV1 mean 6.55±SD 0.61 log_10_ per ml of serum, and TTSuVk2a mean 5.54±SD 0.67 log_10_ per ml of serum) and older animals (TTSuV1 mean 6.29±SD 0.83 log_10_ per ml of serum, and TTSuVk2a mean 5.23±SD 0.84 log_10_ per ml of serum) (p>0.05).

## Discussion

To our knowledge, this is the largest study ever published on the presence of TTSuV in healthy pigs in Italy. More than seven hundred non-consecutive sera were examined for TTSuV DNA using species-specific PCR, and quantified by real time-qPCR. Sardinia is an Italian region at intensive pig farming for commercial use; however, some of these animals are imported from other Italian regions, and some were born abroad (Europe) and imported after weaning. Therefore, our study was scarcely influenced by the ethnic and local origin of the animals, but it is nonetheless important as it well represents the national situation, for which little is known. To date there is only one paper that investigated TTVsuV natural infection in Italy, but it was carried out on a much smaller number of animals and using assays unable to discriminate all TTSuV species [13]. Our study showed that slaughter-age pigs were commonly infected with different TTSuV species, TTSuV1 and k2a species were the most prevalent, and that co-infection with multiple species was fairly common. The data confirm the high prevalence of natural infection with TTSuV in pigs [15], [18], [19]. Interestingly, TTSuVk2b, the most recently identified species, has been also detected in Sardinian pigs, but at a lower frequency compared to other countries [14]. Co-infection with two and/or three species was significantly higher than infections with single species, TTSuV1+TTSuVk2a double infection as the prevalent combination, followed by TTSuV1+TTSuVk2a+TTSuVk2b triple infection. This could be of particular interest, since, similarly to human TTV infection the simultaneous presence of multiple related but distinct TTSuV species and strains has been suggested to favor immune evasion and the establishment of persistent infections [33]. The global prevalence of infection was also compared between genders: similarly to the TTV infection in humans no differences were found [34], [35]. Likewise, the TTSuV prevalence did not vary with age, except for single and triple infections with TTSuVk2b that were more frequent in younger animals (<1 year-old).

Quantitation of positive samples with species-specific real time-qPCR assays evidenced a wide range of viral titers within the species, with the highest levels in TTSuV1 subgroup, and the lowest in TTSuVk2b-positive samples, as previously reported [14]. It would be interesting to investigate if it was due to some sort of competitive advantage between TTSuV species or if TTSuVkb2 has been introduced more recently to Sardinia. Unfortunately, due to lack of previous extended investigation on the circulation of viral species in Italian conventional pigs, we currently cannot infer about the manner and the time in which the k2b species was introduced in the region. Anyway, we are planning to develop an *in* *vitro* system to investigate the possibility that the different viral species can compete each other.

Dynamics of TTSuV DNA load in serum of healthy and sick pigs have been poorly studied until now, but it has been suggested that TTSuV infection could worsen pre-existing or subsequent infections with other pathogens, mainly with porcine circovirus type 2 (PCV2), the causative agent of post-weaning multisystemic wasting syndrome (PMWS) in pigs. In particular, a recent study showed that TTSuV2, but not TTSuV1 DNA load increased over time in animals affected by PMWS [25]. Moreover, TTV DNA load in infected humans appears to correlate with the level of immune competence of the host [36], [37]. Furthermore, a recent study shows that the expansion of members of *Anelloviridae* in human microbiome, and the increasing of viral load in plasma during immunosuppressive therapy could be used to predict and check the immune competence [38]. If this also concerns the animals is an aspect that deserves further investigation. Although animals examined in this study were apparently healthy, we observed that multiple infections with distinct TTSuV species significantly affect viral DNA load and, specifically, that co-infection with TTSuVk2a increases the viral titers of TTSuV1, and co-infection with TTSuVk2b increases the TTSuVk2a DNA load. It would not be surprising if it were also confirmed in animals with other infections and diseases.

Finally, the contribution of animal AVs to the epidemiology of TTVs in humans remains a matter of debate, especially for swine AVs. For this reason, to investigate interspecies transmission of infection and the dynamics of genetic evolution of the virus in humans we started to collect and analyze samples of farms and slaughterhouses employees who have been in contact with the infected animals examined in this study.
